# The Communication of Culturally Dominant Modes of Attention from Parents to Children: A Comparison of Canadian and Japanese Parent-Child Conversations during a Joint Scene Description Task

**DOI:** 10.1371/journal.pone.0147199

**Published:** 2016-01-29

**Authors:** Sawa Senzaki, Takahiko Masuda, Akira Takada, Hiroyuki Okada

**Affiliations:** 1 Department of Human Development and Psychology, University of Wisconsin-Green Bay, Green Bay, Wisconsin, United States of America; 2 Department of Psychology, University of Alberta, Edmonton, Alberta, Canada; 3 Graduate School of Asian and African Area Studies, Kyoto University, Kyoto, Japan; 4 Graduate School of Brain Sciences, Tamagawa University, Tokyo, Japan; Centre for Coevolution of Biology & Culture, University of Durham, UNITED KINGDOM

## Abstract

Previous findings have indicated that, when presented with visual information, North American undergraduate students selectively attend to focal objects, whereas East Asian undergraduate students are more sensitive to background information. However, little is known about how these differences are driven by culture and socialization processes. In this study, two experiments investigated how young children and their parents used culturally unique modes of attention (selective vs. context sensitive attention). We expected that children would slowly learn culturally unique modes of attention, and the experience of communicating with their parents would aid the development of such modes of attention. Study 1 tested children’s solitary performance by examining Canadian and Japanese children’s (4–6 vs. 7–9 years old) modes of attention during a scene description task, whereby children watched short animations by themselves and then described their observations. The results confirmed that children did not demonstrate significant cross-cultural differences in attention during the scene description task while working independently, although results did show rudimentary signs of culturally unique modes of attention in this task scenario by age 9. Study 2 examined parent–child (4–6 and 7–9 years old) dyads using the same task. The results indicated that parents communicated to their children differently across cultures, replicating attentional differences among undergraduate students in previous cross-cultural studies. Study 2 also demonstrated that children’s culturally unique description styles increased significantly with age. The descriptions made by the older group (7–9 years old) showed significant cross-cultural variances in attention, while descriptions among the younger group (4–6 years old) did not. The significance of parental roles in the development of culturally unique modes of attention is discussed in addition to other possible facilitators of this developmental process.

## Introduction

Through interaction with more experienced members of the culture, such as their parents, children learn how to think, perceive, talk, remember, and learn in a given cultural milieu [1–7]. Human parents, different from nonhuman species, actively provide their children with opportunities for cultural learning, skill acquisition, and knowledge acquisition [8–11]. Although cross-cultural variations in perception and attention are well documented in the adult literature, little is known about how children learn such culturally unique modes of attention. By targeting cultural variations in modes of attention between East Asian and North American cultures, the current paper attempts to investigate how parents communicate to their 4- to 9-year-old children in culturally unique modes of attention.

### Cultural Diversity in Modes of Attention in Adults

In the past few decades, accumulating evidence has demonstrated systematic cultural variations in people’s modes of attention, showing that adult members of East Asian societies (mainly Chinese, Japanese, and Korean) are more likely than their Western and North American counterparts (mainly Americans, Canadians, and Western Europeans) to be context sensitive, and to allocate their attention not only to focal objects but also to contextual information (e.g., [12–13]). Cultural variation in attention has been replicated using a variety of cognitive and perceptual tasks such as the change blindness task [14], emotion recognition tasks [15–17], scene perception tasks [12, 18–19], the rod-and-frame task [20], and the framed-line test [21]. For example, Masuda and Nisbett [12] have shown that when American and Japanese undergraduate students were asked to watch and describe short animated vignettes depicting underwater scenes, American students had a tendency to refer to the focal objects, such as large fish, whereas Japanese students had a tendency to describe both the focal objects and the contextual information, such as type of aquatic environment (e.g., lake or ocean). Senzaki et al. [19] replicated these findings with an eye tracker, and further demonstrated that compared to Japanese students, Canadian students spent more time looking at the area where the large fish were swimming, while Japanese students looked at the background area longer than did Canadian students. This confirmed that patterns of eye movements during scene observation corresponded to the verbal description that participants gave after watching the vignettes.

Nisbett and his colleagues speculated that such cultural variation originated in divergent worldviews (a set of culturally shared meaning systems regarding the essential nature of the world and how phenomena are understood), which have been historically developed and sustained by different societies and cultures [22–23]. Nisbett and colleagues suggested that people in European and North American cultures, influenced by ancient Greek philosophical traditions such as Aristotelian logic, share the worldview that objects exist independently from one other. According to this analytic worldview, to better understand a phenomenon, one should carefully attend to the focal issues, events, people, or objects in a scene (i.e., the object-oriented mode of attention). In contrast, people in East Asian cultures, influenced by ancient Chinese philosophical traditions, share the holistic worldview that objects are by nature interrelated. Historically, this worldview has resulted in developing the context-sensitive mode of attention, in which people allocate their attention to the scene as a whole [13, 19]. Similar investigation has also been conducted with people of different socio-ecological backgrounds. For example, hunters and herders have shown to have more analytic or field independent patterns of attention than farmers and fishermen [24–26].

### Gradual Development of Culturally Dominant Modes of Attention in Children

A substantial body of literature in developmental research has demonstrated that sustained visual attention improves considerably from early to middle childhood [27]. Findings in cultural psychology further suggest that although children across cultures may demonstrate similar general abilities to sustain their attention, the type of information they attend to may differ across cultures (e.g. [28]). However, the results are mixed regarding when children begin to demonstrate and reach adult like culturally unique modes of attention (See Table 1). While some evidence suggests that cultural variations in cognition and perception emerge before age 6 [29–35], other studies have demonstrated that, if the tasks involve social judgment, verbal descriptions, and advanced reasoning, such a difference in modes of attention emerge gradually during middle childhood [28] and fully stabilize when the children reach late elementary school [36–38].

**Table 1:** Recent Cross-Cultural Findings in Children’s Development of Attention.

Emergence of Significant (or Adult Like) Cross-Cultural Differences	Tasks	Authors
4	Relational match-to-standard	Kuwabara & Smith (2012)
4	Visual search	Kuwabara & Smith (2012)
4	emotional matching	Kuwabara, Son, & Smith (2011)
4–5	Descriptive accounts	Imada, Carlson, & Itakura (2013)
4–6	coloring	Ishii, Miyamoto, Rule, & Toriyama (2014)
6	Framed-Line Test	Duffy, Toriyama, Itakura, & Kitayama (2009)
6–7	First mentioned objects	Imada, Carlson, & Itakura (2013)
6–7	Ebbinghaus illusion	Imada, Carlson, & Itakura (2013)
7–9 (Second Grade)	drawing a horizon	Senzaki, Masuda, & Nand (2014)
10	Emotion judgement	Masuda et al. (2015)
11	Prediction of change	Ji (2008)
11–15	Social attribution	Miller (1984)

For example, Masuda et al. [37] presented cartoon images of lineups of five children including a central figure and background figures which sometimes showed the same emotions (e.g., happiness) and sometimes showed incongruent emotions (e.g., happy central figure and sad background figures). Masuda and colleagues asked both Japanese and Canadian children to judge the central figure’s emotion and later reason why they perceived it that way. The results indicated that Japanese children’s attention gradually became more context sensitive with age. That is, as Japanese children’s age increased, their judgment was more likely to be influenced by contextual information (e.g., accentuating the intensity of central figure’s emotions in congruent vs. in incongruent conditions) and their reasoning was more likely to refer to the social context. Cross-cultural differences in the explanation styles increased with age, as Canadian children in all age groups from 7 to 10 tended to selectively focus on the central figure. Accordingly, a substantial cross-cultural difference appeared at age 10. Similarly, Ji [36] examined when children demonstrate cross-cultural differences on the perception of change. She asked 7- to 11- year-old Chinese and Canadian children to predict future performances and relationships (e.g., whether good friends continue to be good friends or the relationship falls apart in the near future), and demonstrated that, at age 11, Chinese children were more likely than their Canadian counterparts to predict changes. Miller [38] also demonstrated that, when asked to explain possible causes of an event, Indians had a tendency to refer to external factors (e.g., social constraints) whereas Americans tended to refer to internal factors (e.g., personality), and these cultural difference became significant at ages 11–15.

Thus far, the results of research on culture and development converge to provide accumulating evidence demonstrating that children gradually learn culturally dominant modes of attention, rather than at a specific age. Based on these findings, we began the current studies by identifying tasks by which children would demonstrate the presence of early signs of cultural variations in attention, but before they fully demonstrate significant cross-cultural differences. By pinpointing a task that is slightly beyond the children’s current degree of internalization of a culturally shared perspective, we expected that parents would have a significant role in fully stabilizing children’s culturally dominant modes of attention.

### Parental Influence on Children’s Attention Styles

While examining children’s own development, there is an indispensable issue that has not been examined fully in the context of research on attention. That is, what factors influence the development of children’s internalization of beliefs shared by members of a given culture and reflected in modes of attention? This issue has been the focus of interdisciplinary discussions as it would be one of the most important factors to understand human sociality. For example, in the discourse of cultural evolution, this issue has been discussed as “cultural transmission” [8, 39–40, 7, 11]. In the discourse of developmental sciences, caregivers’ roles in the transmission process have been actively discussed under the rubrics of the zone of proximal development [9, 41–42] and scaffolding [10, 43–44], indicating that mature members of a culture (e.g., parents) provide temporal support for children in order to facilitate learning of emerging skills. Previous research has demonstrated that children’s preferences in storybooks [45] and how they color shapes [30] correspond to societal and parental preferences, suggesting that the meaning systems of mature members of a given society influence children’s cognitive and perceptual tendencies. Furthermore, empirical studies have shown how joint cognitive activities between children and adult partners foster children’s cognitive development [46–52].

Empirical findings of parents’ behaviors with children suggest that parents exhibit culturally dominant behaviors to children even when children are still too young to demonstrate such behavioral patterns. For example, Fernald and Morikawa [53] demonstrated that, when interacting with their infants, American mothers tended to direct infants’ attention to the target toy by referring to its focal attributes such as color and shape. On the other hand, Japanese mothers tended to refer to the relationships between the toys, the mother, and the infant while directing the child’s attention to multiple objects and persons in the scene. Similarly, Wang and her colleagues [54] studied how mothers interacted with their 3-year-old toddlers while reading a children’s book and found that the conversations of American mother–child dyads revolved more around the independent view of self, whereas Chinese mother–child conversations emphasized the interdependent view of self in the context of social others. Masuda and his colleagues [37] also demonstrated that, when Japanese and Canadian parents were asked to perform a social judgment task in front of their children (7- to 8-years-old), parents exhibited culturally unique modes of attention even though the children did not demonstrate such cultural differences. Based on the previous research, we expected that children’s cognitive development would be embedded in such social interactions with mature members of a given culture. Interactions among parents and children would thus be a source of rich and valuable opportunities for parents to transmit their culturally unique mode of attention to their children, and for children to integrate their communication style with their model’s (parent’s) mode of attention.

### Overview of Studies

Our main goal was to investigate how parents communicate to their children in culturally dominant modes of attention before their children demonstrate a stabilized internalization of such culturally unique patterns of attention. With the intent to examine 1) children’s attention during solitary performances, and 2) the role of parental support for children in learning culturally unique modes of attention, we conducted two cross-cultural studies using a memory-based free description task [12, 19]. This task asks participants to observe animation vignettes of underwater scenes, and freely describe the scenes based on their memory. This task has reliably demonstrated cultural differences in modes of attention between North American and East Asian undergraduate students.

Previous studies have shown that by the age of 4, children from North American and East Asian cultural groups demonstrated evidence of cultural differences in attention, yet it was not until age 10 that systematic cultural differences between these groups were fully stabilized in complex tasks (e.g., [37]). Given our interest in capturing preceding sources of cultural differences in children’s attention, we focused on the ages in which children are learning culturally dominant modes of attention. Therefore, we chose participants between the ages of 4 and 9, assuming that culturally unique modes of attention have begun to gradually develop by this point, although internalization of such modes of attention has not yet been achieved. Accordingly, this age group would be ideal for examining the parental influence on children’s task performance. Our preliminary observation also confirmed that children were able to follow the directions provided in order to complete the memory-based free description task [12, 19] at the age of 4. Age-related changes in children’s performance were investigated by comparing younger (ages 4–6) and older (ages 7–9) children. We assumed that older children would more likely to be influenced by their parents than younger children, because previous research has indicated children become more sensitive to their parents’ communicative cues particularly at around age 7. For example, children’s ability to perceive another person’s perspective develops gradually beginning around age 4, and the ability to become less egocentric and to understand others’ viewpoints increases dramatically at around age 7 [55]. These abilities are important for children to have when jointly engaging cognitively challenging tasks with their parents. The significance of these abilities was demonstrated in a study where children engaged in a visual search task with their parents. In this study, only 7- to 8-year-old children, and not 4- to 5-year-old children were able to demonstrate the effect of learning from their parents [56]. Thus, in our study we divided the participants into the following two age groups in order to effectively compare their performance: 4- to 6- and 7- to 9-year-olds.

In Study 1, we tested the appropriateness of the task. We hypothesized that younger and older children (4- to 9-year-olds) would not yet demonstrate significant cross-cultural differences when they completed the task by themselves. In Study 2, we used the same task in order to examine how parental intervention during the task would help children attain culturally unique modes of attention. We hypothesized in Study 2 that parents would constantly apply culturally dominant modes of attention (i.e., object oriented attention in Canada and context-oriented attention in Japan) while engaging in the task with their children regardless of their child’s age. We also hypothesized that older children (7–9 years old), but not younger children (4–6 years old), would be influenced by their parents, and subsequently exhibit culturally dominant modes of attention (Study 2).

## Study 1: Preliminary Study

### Participants

Seventy (39 girls, 31 boys) 4- to 9-year-old (*M* = 6 years 7 months, *SD* = 1 year 8 months) Canadian children were recruited in Edmonton, Alberta. A comparable sample of 71 (38 girls, 32 boys), 4- to 9-year-old (*M* = 6 years 6 months, *SD* = 1 year 9 months) Japanese children in Kyoto and Tokyo, Japan participated in the study. Among Canadian participants, 62 children (89%) were European-Canadian, 1 child (0.01%) was Hispanic, 1 child (0.01%) was African-Canadian, and 6 (0.09%) children were of mixed ethnicity. All Japanese children were born and raised in Japan. Both samples were predominantly upper middle class but we did not exclude participants based on their social economic status. In both Canada and Japan, over 90% of parents had a college degree or higher, and the distribution in each culture was compatible, χ^2^ = 0.89, *ns*. Parents’ educational levels were not significantly correlated with any of the variables of present interest and thus were omitted from further analysis. Participating children’s parents gave us written consent and children gave us verbal assent to participate in the study.

In the analyses, participants were divided into two age groups. Younger children were 4- to 6-year-old (32 Canadian: *M* = 5 years 2 months, *SD* = 10.9 months; 33 Japanese: *M* = 4 years 10 months, *SD* = 11.8 months), and older children were 7- to 9-year-old (38 Canadian: *M* = 7 years 11 months, *SD* = 12 months; 38 Japanese: *M* = 7 years 10 months, *SD* = 11 months). Both in Canada and Japan, all the children were either in pre-school or in school. Genders of children did not influence the results, and therefore, genders were collapsed in the further analyses.

Testing was conducted either in a laboratory space at the universities or in a quiet room at either the local daycare or after-school program. We made the settings of data collection sites as similar as possible across the two countries. The study was approved by the Research Ethics Board at the University of Alberta (file Number of the current study: Pro00009915).

### Materials and Procedure

**Fig 1:**
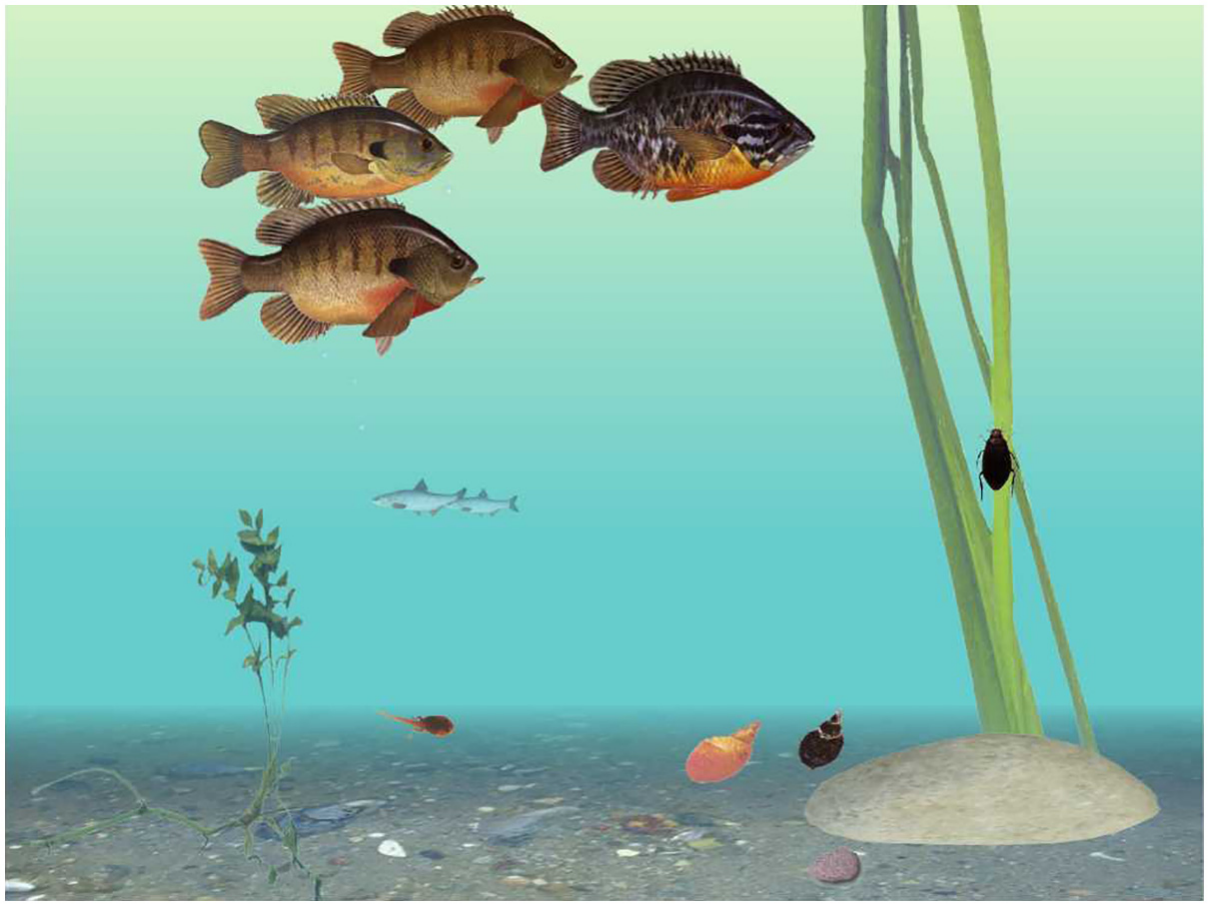
Sample experimental stimulus.

We modified six animated vignettes of underwater scenes originally created by Masuda and Nisbett [12] by adding background water sounds, which were intended to sustain children’s motivation to complete the task (see Fig 1). There were no statistical differences in response patterns across six vignettes; therefore we treated them equally, and used the average value of the descriptive accounts. Each animation lasted approximately 25 seconds and included different types of fish, small sea animals, and background objects, as well as different types of scenery with various colors, such as green lake and blue ocean underwater scenes. Using the same computer in both cultures, vignettes were presented on a laptop computer with a 15.4-inch display using Adobe Flash. All the instructions were translated and back-translated from English to Japanese (for a review of the translation method, see [57]), and the task was administrated in English for Canadian participants and in Japanese for Japanese participants.

After warm-up play activities which lasted for 5 to 10 minutes, the child was asked to watch the animation vignettes and describe the content in as much detail as possible at the end of each vignette. Four vignettes were randomly selected, and as in previous research [12], each vignette was played twice. When the animation vignette ended, the experimenter prompted the child to describe his or her observation freely based on memory, and ensured the child that there were no right or wrong answers. This procedure followed the instruction used in previous research by Imada et al. [28]. The experimenter asked questions such as “Is there anything else?” or “Can you tell me anything else?” until the child indicated that there was nothing more to describe. The same procedure was repeated for each vignette, and the memory-based verbal description was combined across two viewings of the same vignette and averaged across 4 vignettes for the analyses.

Sessions were videotaped and transcribed afterwards. The child’s memory-based verbal descriptions were used as a measure of attention. Although this is an indirect measure, previous research suggests that verbal description is a valid measure of attention because there was a strong association between memory-based verbal descriptions made by participants and their patterns of eye movements [19].

### Coding

Adopting a coding schema established in previous research [12, 19] we divided transcribed data into the smallest descriptive accounts, which were used as the coding units. Each account was primarily categorized into three groups: focal objects (including information regarding focal fish and background fish), background (including inert living beings such as shellfish and stationary things such as plants and rock formations, as well as other environmental information such as type of water body), and active living beings (including moving objects such as frogs or bugs that are not the main characters in the scene). For example, when the participant described the animated vignette as “I saw two large fish,” the meaningful accounts that described the animated vignette were “*two”* (the number of the referred target), “*large*” (the attribute of the referred target), and “*fish*” (the target categorized as the focal object); thus, this description was coded as three accounts for focal objects. These coding categories were mutually exclusive. We did not expect to see any cross-cultural differences with the active living being category because they were neither main objects nor background. As the analyses were based on the units of meaningful accounts, this coding schema minimized the syntactical differences between English and Japanese languages (for further discussion, see [19]).

All data coding was performed in the original languages. Two English–Japanese bilingual coders who were blind to the hypotheses independently segmented and coded both Canadian and Japanese data, and intercoder agreements for the overlapping 25% of the data set were .91 and .93 for Canadian and Japanese data respectively. Disagreements were resolved by discussion between coders, and the first author made the final decisions.

## Results and Discussion

To examine age-related cognitive development, we first examined the total number of accounts (see S1 Data and S1 Text). As we expected, the total number of children’s descriptive accounts increased with age, *F*(1, 137) = 35.82, *p* = .001, η_p_^2^ = .20. There was no effect of Culture, *F*(1, 137) < 1, *ns*, nor interaction between Age and Culture, *F*(1, 137) < 1, *ns*. As we expected, there was a universal pattern in children’s modes of attention, such that both older Canadian (*M* = 11.16, *SD* = 10.91) and Japanese (*M* = 10.50, *SD* = 8.32) children made a larger number of accounts in general than younger Canadian (*M* = 5.70, *SD* = 4.84) and Japanese (*M* = 5.92, *SD* = 5.38) children. It was thus necessary to control for these age-related differences in communication ability (e.g., [28]) to clearly demonstrate the target information. Accordingly, we used the total number of descriptive accounts as a covariate in the following analyses.

First, we examined attention to the focal objects. A 2 (Culture: Canada, Japan) × 2 (Age Group: 4–6, 7–9 years) analysis of covariance (ANCOVA) was conducted on children’s accounts of focal objects. As expected, there was no effect of Culture, *F*(1, 136) = 2.40, *p* = .12, Age, *F*(1, 136) = 2.57, *p* = .11, nor interaction between them, *F*(1, 136) < 1, *ns*. After controlling for children’s total accounts, the mean accounts of the focal objects were similar across all the groups: younger Canadian (*M* = 10.13, *SD* = 4.98), older Canadian (*M* = 8.28, *SD* = 4.85), younger Japanese (*M* = 8.47, *SD* = 4.81), and older Japanese (*M* = 7.45, *SD* = 4.87) children.

Next, we examined attention to the background. Again, a 2 (Culture) × 2 (Age Group) ANCOVA revealed no effect of Culture, *F*(1, 136) = 1.22, *p* = .27, Age, *F*(1, 136) = 2.55, *p* = .11, nor interaction between them, *F*(1, 136) < 1, *ns*. After controlling for children’s total accounts, the mean accounts of the background were similar across all the groups: younger Canadian (*M* = 8.53, *SD* = 4.96), older Canadian (*M* = 10.18, *SD* = 4.83), younger Japanese (*M* = 9.63, *SD* = 4.79), and older Japanese (*M* = 10.84, *SD* = 4.85) children.

Finally, we examined attention to the active living beings. As we expected, a 2 (Culture) × 2 (Age Group) ANCOVA revealed no effect of Culture, *F*(1, 136) = 1.85, *p* = .17, Age, *F*(1, 136) < 1, nor interaction between them, *F*(1, 136) < 1, *ns*. After controlling for children’s total accounts, the mean accounts of the background were similar across all the groups: younger Canadian (*M* = 5.53, *SD* = 4.86), older Canadian (*M* = 5.95, *SD* = 4.37), younger Japanese (*M* = 6.64, *SD* = 4.45), and older Japanese (*M* = 6.27, *SD* = 4.54) children.

The results of Study 1 support our first hypothesis and confirm that the current task was indeed slightly above the children’s cultural patterns of attention. Although there were non-significant tendencies of cross-cultural variation, 4- to 9- year-old Canadian and Japanese children did not demonstrate significant cross-cultural differences in their modes of attention while completing the current memory-based scene description task by themselves. Therefore, the results confirm that the measure is appropriate for investigating the issue addressed in Study 2.

## Study 2: Main Study

Study 2 aimed to examine the performance of 4- to 9-year-old children and their parents in Canada and Japan during the memory based free-description task. There were two phases in Study 2. First the children were asked to describe their observation of animated vignettes alone (Phase 1: Solitary description condition) and then together with their parents (Phase 2: Joint description condition). We coded and analyzed the content of both parents’ and children’s verbal descriptions of the scenes as a measure of culturally varying modes of attention. We expected that parents would demonstrate significant cross-cultural differences in their descriptive accounts while jointly engaging in the task with their children. Specifically, we hypothesized that Canadian parents would be more likely to attend to focal objects than Japanese parents, while Japanese would be more likely to attend to the background than Canadian parents. We also tested whether or not, and when children would demonstrate cultural learning in this parent-child dyadic condition. We expected that when compared to the children’s solitary performance in which children complete the task by themselves, children would be more likely to remember and talk more overall when jointly completing the task with their parents. As a result, we hypothesized that older children would show cross-cultural differences in their attentional modes when they completed the task jointly with their parents. On the other hand, we expected that younger children would not yet show cultural differences in patterns of attention as they had not yet reached the developmental level at which they would be able to take advantage of parents’ inputs.

## Method

### Participants

Participants were again 4- to 9-year-old children and their parents. In total, the data of 107 parent–child dyads (50 dyads in Canada and 57 dyads in Japan) were collected. The Canadian children consisted of 27 girls and 23 boys, and the Japanese children consisted of 30 girls and 27 boys. Two additional children did not complete the task, and the data from three other dyads were excluded because of a recording error. Canadian parent–child dyads were recruited via a research participant database at the University of Alberta and flyers posted in local daycares and after-school programs in Edmonton, Alberta. One of the Canadian children who completed the study was born in an Asian country and was adopted by a Canadian family when she was 2.5 years old. All other parents in Canada identified themselves and their children as Caucasians of European descent. Japanese parent–child dyads were recruited via existing research participant databases at Kyoto University and at Tamagawa University. All Japanese parents and their children were born and raised in Japan. In both countries, all parent–child pairs came from the middle or upper middle class, and the level of parents’ education was similar across both cultural groups (95.6% of Canadian parents and 89.3% of Japanese parents had a college degree or higher, χ^2^ = 1.64, *ns*). Parents’ educational levels were not significantly correlated with any of the variables of present interest and thus were omitted from further analysis. The gender distribution among participating parents was as follows: 5 fathers and 45 mothers in Canada, and 3 fathers and 54 mothers in Japan.

As in Study 1, participants were divided into two age groups of children. Younger children were 4- to 6-year-old (26 Canadian: *M* = 5 years 2 months, *SD* = 8.7 months; 29 Japanese: *M* = 5 years 2 months, *SD* = 8.5 months), and older children were 7- to 9-year-old (24 Canadian: *M* = 7 years 11 months, *SD* = 11.5 months; 28 Japanese: *M* = 7 years 10 months, *SD* = 10.5 months). Genders of parents and children did not influence the results, and therefore, genders were collapsed in the further analyses.

### Materials and Procedure

We used exactly the same material as in Study 1. Two vignettes were used in Phase 1 (solitary description condition) and four vignettes were used in Phase 2 (joint description condition) in random order. The task was again administrated in English for Canadian participants and in Japanese for Japanese participants. Upon arrival, the parent completed a consent form, while the child engaged in warm-up play activities for 5 to 10 minutes. We received a written consent from parents on behalf of themselves and their children. We received verbal assent from children. The child first completed the task alone (Phase 1), and then both parent and child completed the task together (Phase 2). The procedure of Phase 1 was the same as Study 1. While the children engaged in the solitary description task in Phase 1, the parent completed a demographic questionnaire in the same room.

After the child’s solitary description condition, the parent joined the session in Phase 2. There, both child and parent were asked to jointly engage in the scene description activity. In this phase, child and parent sat next to each other in front of a computer monitor. Again, the parent and child were told that there were no right or wrong answers, and they freely described the content of the animation vignette in as much detail as possible at the end of each animation vignette based on memory after each vignette was presented. They were also instructed to talk naturally just as they would when they were at home watching TV or reading a book together. Four animated vignettes were played in random order in Phase 2, and each vignette was played twice. To measure modes of attention, we coded verbal descriptions given by parents and their children in the same way as in Study 1.

## Results and Discussion

As in Study 1, we first examined the overall volume of accounts. Two variables were used to measure the overall volume of memory-based free description: a) the total number of accounts made my children and parents coded separately, and b) the number of conversational turns made by parents and children.

### Total volume

To test the general trend of age-related cognitive development and the effect of condition (solitary vs. joint), we conducted a 2 (Culture: Canada, Japan) × 2 (Age Group: 4–6, 7–9 years) × 2 (Condition: Solitary, Joint) ANOVA with culture and age group as between-subject factors and condition as a within-subject factor on the total number of accounts (see supplementary documents for the complete data set). There were main effects of Age, *F*(1, 103) = 106.03, *p* = .001, η_p_^2^ = .51, and Condition, *F*(1, 103) = 73.42, *p* = .001, η_p_^2^ = .42. There was also an interaction effect between Age and Condition, *F*(1, 103) = 10.12, *p* = .01, η_p_^2^ = .09. Both younger and older children made a larger number of accounts in the joint condition (younger Canadian: *M* = 9.44, *SD* = 6.94; younger Japanese: *M* = 8.88, *SD* = 5.75; older Canadian: *M* = 27.98, *SD* = 15.24; older Japanese: *M* = 22.80, *SD* = 13.32) than in the solitary condition (younger Canadian: *M* = 14.34, *SD* = 5.46; younger Japanese: *M* = 16.64, *SD* = 9.67; older Canadian: *M* = 34.16, *SD* = 17.26; older Japanese: *M* = 38.80, *SD* = 16.48). There was no cross-cultural difference on the number of total number of accounts.

Next, we examined the number of total accounts made by parents as a function of children’s age and culture. A 2 (Culture) × 2 (Age Group) ANOVA on parents’ total number of descriptive accounts revealed that children’s age was a significant factor, *F*(1, 103) = 9.81, *p* = .01, η_p_^2^ = .09. Parents of older children in Canada (*M* = 42.23, *SD* = 22.87) and Japan (*M* = 39.01, *SD* = 21.52) made a larger number of accounts than did parents of younger children in Canada (*M* = 29.21, *SD* = 13.52) and Japan (*M* = 28.86, *SD* = 17.28). There was no cross-cultural difference on the number of parents’ total descriptive accounts, *F*(1, 103) < 1, *ns*. Again, in order to control for these age-related differences, we used the total number of descriptive accounts as a covariate in the following analyses.

We also examined the total number of conversational turns taken by parents and children. A 2 (Culture: Canada, Japan) × 2 (Age Group: 4–6, 7–9 years) ANOVA on the number of conversational turns revealed that older parent-child dyads took more conversational turns (Canadian: *M* = 58.75, *SD* = 26.57; Japanese: *M* = 69.36, *SD* = 32.46) than younger parent-child dyads (Canadian: *M* = 38.69, *SD* = 16.79; Japanese: *M* = 41.07, *SD* = 18.31), *F*(1, 103) = 26.21, *p* = .001, η_p_^2^ = .20. There were no significant main or interaction effects of culture on the number of conversational turns taken by parents and children.

In each of the following main analyses, we examined two types of attention: attention to the focal objects and attention to the background. Based on previous research with adults, we expected that Canadian participants in general would attend more to the focal objects than would Japanese participants, and Japanese participants would attend more to the background than would Canadian participants. In the following, we first present results from parents’ description in the joint description activity (Phase 2), and then we present the results of children’s behaviors and how they shift from the solitary description (Phase 1) to the joint description (Phase 2).

### Parents’ Verbal Description During the Joint Report Phase

#### Focal objects

**Fig 2:**
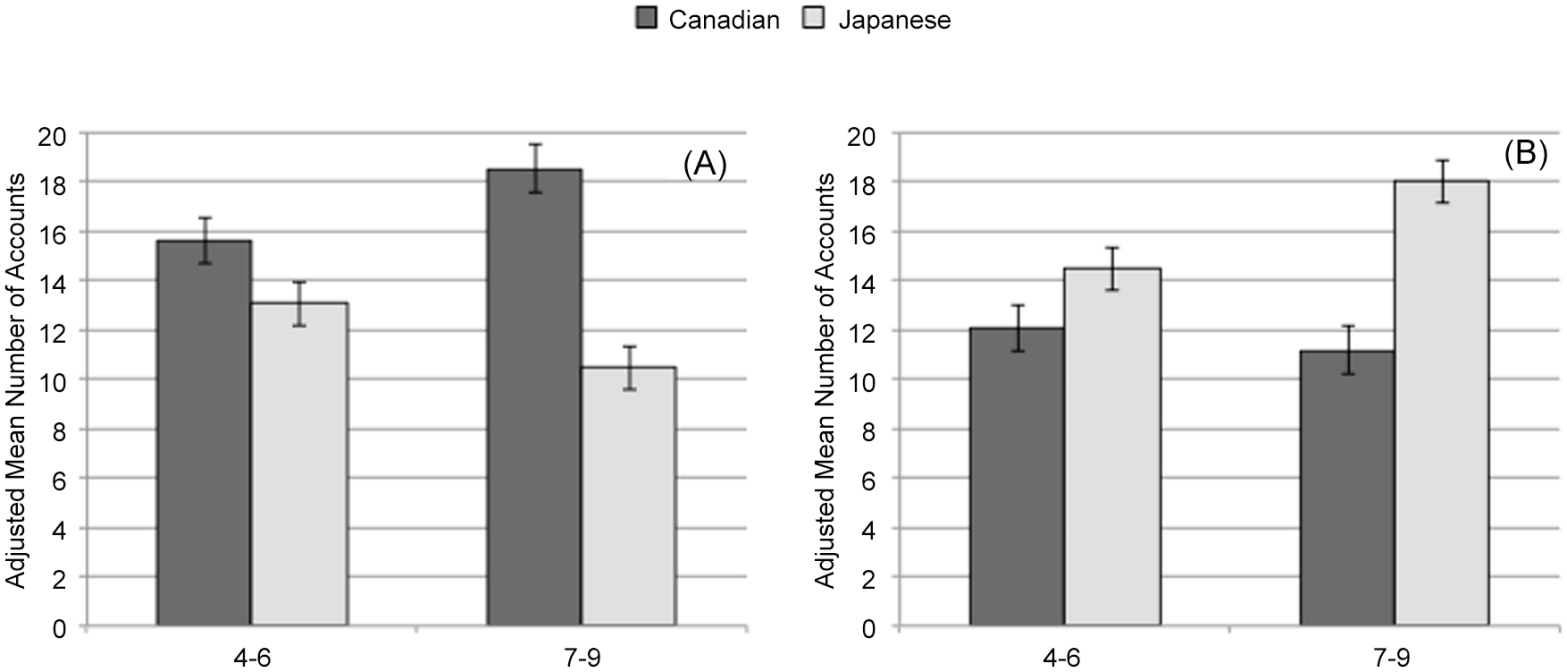
Parents’ attention to the focal objects and background by culture and children’s age. (A) Parents’ attention to the focal objects measured by adjusted mean number of descriptive accounts during joint description. (B) Parents’ attention to the background measured by adjusted mean number of descriptive accounts during joint description. Error bars represent standard errors.

Using parents’ total accounts as a covariance, a 2 (Culture: Canada, Japan) × 2 (Age Group: 4–6, 7–9 years) analysis of covariance (ANCOVA) on parents’ accounts of focal objects yielded a significant main effect of Culture, *F*(1, 107) = 33.78, *p* = .001, η_p_^2^ = .25. As shown in Fig 2A, Canadian parents (*M* = 17.09, *SD* = 4.95) referred to the focal objects significantly more than Japanese parents did (*M* = 11.77, *SD* = 4.50), after adjusting for parents’ total account. More interestingly, this main effect of culture was qualified by the significant interaction with Age Group, *F*(1, 107) = 8.99, *p* = .01, η_p_^2^ = .08, because there was significant increase in effect sizes for cultural differences with age of children (η_p_^2^s for 4–6, 7–9 = .08 and .41, respectively).

#### Background

As shown in Fig 2B, a 2 (Culture) × 2 (Age Group) ANCOVA on parents’ accounts of the background revealed a significant main effect of Culture, *F*(1, 107) = 26.83, *p* = .001, η_p_^2^ = .21. After controlling for parents’ total accounts, Japanese parents (*M* = 16.26, *SD* = 7.21) in general referred to the background information significantly more than did Canadian parents (*M* = 11.62, *SD* = 7.42), which was qualified by a significant Culture × Age Group interaction, *F*(1, 107) = 6.14, *p* = .05, η_p_^2^ = .06. Again, there was significant increase in effect sizes for cultural differences with age of children (η_p_^2^s for 4–6, 7–9 = .08 and .33, respectively).

#### Active Living Beings

Finally, as we expected there was no effect of Culture, *F*(1, 107) = 2.15, *p* = .15, nor interaction between Culture and Age Group, *F*(1, 107) = 1.28, *p* = .26, on parents’ attention to the active living beings.

### Shift in Children’s Verbal Description

Parents’ guidance during joint description was expected to be a vehicle for children to learn culturally unique ways of processing visual information. Accordingly, we expected that children would be more likely to show cross-cultural differences when they engaged in the description task with their parents (Phase 2), rather than when they engaged in the task alone (Phase 1). In order to examine parental influence on children’s behaviors, we compared children’s behaviors between the solitary condition (Phase 1) and the parent–child joint description condition (Phase 2). As in the previous analyses, we present the findings of focal objects first and background objects second.

#### Focal objects

**Fig 3:**
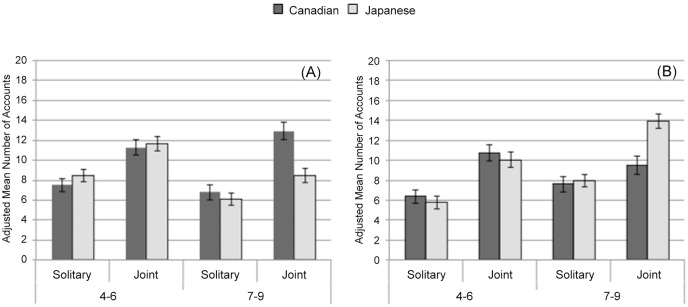
Children’s attention to the focal objects and background by culture, age, and condition. (A) Children’s attention to the focal objects measured by adjusted mean number of descriptive accounts during solitary and joint descriptions. (B) Children’s attention to the background measured by adjusted mean number of descriptive accounts during solitary and joint descriptions. Error bars represent standard errors.

A 2 (Culture: Canada, Japan) × 2 (Age Group: 4–6, 7–9 years) × 2 (Condition: solitary, joint) ANCOVA with Culture and Age Group as between-subject factors and condition as a within-subject factor, controlling for children’s total accounts, revealed a significant 3-way interaction, *F*(1, 101) = 4.60, *p* = .05, η_p_^2^ = .04 (Fig 3A). To explore this Culture × Age Group × Condition interaction, we conducted separate 2 (Culture) × 2 (Condition) ANCOVAs for each age group. The Culture × Condition interaction was significant for older children, *F*(1, 48) = 7.91, *p* = .01, η_p_^2^ = .14, but not for younger children, *F*(1, 51) < 1, *ns*. Planned simple effect analyses among older children revealed that the cross-cultural difference was significant when they engaged in the task with their parents, *F*(1, 49) = 12.92, *p* = .001, η_p_^2^ = .21, but not when they engaged in the task alone, *F*(1, 49) < 1, *ns*. These results indicated that older children’s verbal descriptions changed as a function of condition, showing that Canadian children were significantly more likely to discuss focal objects than Japanese children only when they engaged in the task with their parents.

#### Background

A 2 (Culture) × 2 (Age Group) × 2 (Condition) ANCOVA with children’s total accounts as a covariate on children’s accounts of the background also yielded a significant 3-way interaction, *F*(1, 101) = 7.17, *p* = .01, η_p_^2^ = .07 (Fig 3B). Further analyses revealed that a Culture × Condition interaction was significantly different among older children, *F*(1, 48) = 8.99, *p* = .010, η_p_^2^ = .16, but not among younger children, *F*(1, 51) < 1, *ns*. Planned simple effect analyses among older children demonstrated that children’s verbal description changed as a function of condition, such that cross-cultural differences was significant when older engaged in the task with their parents, *F*(1, 49) = 12.56, *p* = .001, η_p_^2^ = .20, not significant when they engaged in the task alone, *F*(1, 49) < 1, *ns*. Japanese older children were significantly more likely than Canadian older children to discuss background objects only while engaging in the task with their parents.

#### Active Living Beings

Again, children’s attention to active living beings did not differ across cultures, *F*(1, 107) < 1, *ns*, and there were no other interaction effects.

In summary, findings of Study 2 confirmed our hypotheses. First, consistent with the results of Study 1, cultural variations in modes of attention did not reach significance in either age group when children engaged in the task alone in Phase 1. Second, as we expected, the results demonstrated that parents communicated to their children in culturally attuned modes of attention during joint task engagement, and parents of older children exhibited relatively stronger cross-cultural differences than those of younger children. Finally, older children both in Canada and Japan were more likely to contribute to the conversation as indicated by the number of total accounts, and by the number of conversational turns taken by parents and children. All of these factors may have contributed to the age-related differences in children’s behavior during joint description, thereby resulting in only older children demonstrating significant cross-cultural differences in modes of attention while engaging in the task jointly with parents.

## General Discussion

### Summary of Findings

In this paper, we selected the memory-based free description task by adapting Masuda and Nisbett’s (12) original task. In the preliminary study (Study 1), we were able to confirm that this task was an appropriate measure to examine how parents communicate to their children differently across cultures before these children demonstrate such perspectives themselves. The results of Study 1 showed that children did not demonstrate significant cross-cultural differences at 4 to 9 years of age when completed alone, although the trend towards cultural difference was emerging. We then examined how parents conveyed cultural messages, and how children accommodated culturally dominant modes of attention through parent–child interactions in the main study (Study 2).

Overall, the results of the current studies support our hypotheses. First, we identified one culturally universal pattern of cognitive development in children, in that children’s descriptive accounts increased generally with their age. We also identified that 4- to 9-year-old children in our studies did not demonstrate significant cross-cultural differences when completing the memory-based free description task alone, as shown in Study 1 and in Phase 1 of Study 2.

Considering that this task was slightly above the children’s current state of development, it was important to demonstrate that the ways in which the parents communicated to their 4- to 9-year-old children differed significantly across cultures. Specifically, Study 2 demonstrated that Canadian parents made more references to focal objects than did Japanese parents, and Japanese parents made more references to the background than did Canadian parents. These results are consistent with those of previous studies involving undergraduate students [12, 19]. The current studies further add to this literature by demonstrating cross-cultural differences in attention among parents. These studies also provide strong support for the existence of two distinctive modes of attention largely shared by people of two respective cultures: That is the object-oriented mode of attention in Canada and the context-sensitive mode of attention in Japan. The current studies were the first to demonstrate parents communicating to their children with culturally dominant modes of attention. We maintain that our laboratory studies reflect how parents regularly provide opportunities for their children to learn and participate in a meaning system shared in a given culture.

Mirroring their parents’ guidance, Canadian 7- to- 9-year-olds were more likely than Japanese children to discuss focal objects, while Japanese 7- to 9-year-olds were more likely than Canadian children to discuss contextual information. Such cross-cultural differences did not reach significance in 4- to 9-year-old children during the joint description task. In other words, older children were more attuned to their parents’ teaching, and in turn, the cultural communication patterns of parents may have been even stronger when they were responding to older children. Parent and older-child dyads produced a significantly larger number of accounts in total and took a significantly larger number of turns than younger dyads, and these general age-related differences may have contributed to the greater cross-cultural differences seen in older children in our studies.

Given the previous findings on children’s cognitive development [55–56], we did not expect to find substantial cross-cultural differences for our younger participants. Other findings also provide support to age-related differences in children’s cognition. For example, compared to preschool-age children, school-age children are much more likely to use complex cognitive skills to sustain their attention and memory [58–61]. Such abilities related to memory and attention may help older children engage in more complex conversations with their parents, which in turn may help them attune to their parents’ direction. In addition, older children’s advanced development in relation to inferring others’ intentions may help them recognize that their parents, as mature members of the culture, have a message to convey during joint activities [62–64]. Our findings indeed suggest that older children are more attentive to parents’ intentions while attending to the scene in the joint activity, while younger children seem to be less concerned with this.

Another question to be discussed here is the types of tasks that can be used to examine cultural and developmental differences in modes of attention. In studies involving North American and East Asian adults, the current attention task has been reliable in demonstrating systematic cultural variations [12, 19]. Although other researchers have employed different tasks to demonstrate cross-cultural differences in children’s behaviors without parental input at a young age (e.g., [31–32]) children’s behaviors during the solitary description in the current study were not different across cultures in any age group. It is important to note that our measure was more complex than previous measures in which younger children themselves demonstrated significant cross-cultural differences. We maintain that the difference between the current study and prior studies may be attributed to the differences in task difficulty. The current studies used Masuda and Nisbett’s [12] attention task which requires advanced memory-based verbal communication on the part of children, who were asked to remember and describe the animated vignettes in their own words. In contrast, most tasks used in the previous studies did not require such advanced skills while presented with complex information. Although such a memory-based verbal task may not be the best task to identify the earliest onset of cultural differences in children’s cognitive development, we intentionally selected this task in order to examine how parents transmit culturally shared cognitive styles to their children via verbal communication. While the current task was appropriate for examining parent-child interactions, researchers should employ a variety of tasks including varying difficulties if they plan to identify the emergence of cross-cultural differences in children’s solitary performance.

### Implications

The current study has three major implications. First implication of the current study relates to the development of sociocognitive skills. The results of the task can be considered evidence of the joint attention process. Joint attention occurs when two individuals attend to the same object or event together, and both participants monitor each other’s attention to the target of mutual interest while coordinating their own action [7]. Several researchers further maintain that the active engagement in shared activity is an important component to sustain culture and transmit cultural knowledge (e.g. [65]). Although joint attention has been studied primarily with infants (e.g., [66–67]), the current findings demonstrate that joint attention and the active engagement in shared activity facilitate even school-age children’s cognitive development and provide the context in which they are exposed to culturally shared modes of attention. In fact, Bruner [68] emphasized the importance of the joint activities as the source of cognitive development throughout childhood. In line with Bruner’s theoretical assumption, we showed that joint activities provide an important foundation for a child’s engagement in the development of necessary skills for interpreting the environment in a culturally appropriate manner.

Second, previous studies in developmental sciences identify the role of social structures on the development of various modes of attention. For example, cross-cultural research in attention management has demonstrated that both mothers and children from indigenous communities of Mexico are more likely to attend to multiple events simultaneously than are European American mothers and their children, and the differences were interpreted as a result of culturally different social structures, such as the differences in family sizes [69–70], we maintain that culturally shared meaning systems also play a role in shaping children’s development. The current findings suggest that culturally shared meaning systems, such as the holistic vs. analytic thought shared by parents in East Asian vs. North American cultures, could be an alternative source of cultural differences in the development of various modes of attention. We suggest that rather than excluding either one of these explanatory factors, investigating how and when these factors mutually or solely influence the development of modes of attention will provide us with fruitful discussions [71].

Third, the findings of the current studies add to the literature of cultural evolution, particularly in relation to the issues of cultural transmission [7, 8, 11, 39, 40, 72]. Bartlett [73] originally developed a method that resembles the children’s game “Telephone” or “Chinese whispers” to examine how information is passed along a chain of participants. Several empirical studies have applied this method (e.g., [39]), and these studies provide insight applicable to future research on the transmission of culturally shared modes of attention. In particular, research conducted by Kashima and his colleagues [74–76] demonstrated that an existing cultural belief became stronger when that information was transmitted through a line of ingroup members. Imada and Yussen [77] have also demonstrated that narratives played an important role in reproduction of cultural values in a laboratory setting. Particularly, American participants were more likely to create stories and retained information based on individualistic values, while Japanese participants were more likely to create stories and retain information based on collectivistic values. The current findings in our studies can be interpreted in light of cultural transmission occurring between an experienced individual (parent) and a novice learner (child). In Study 2, culturally shared patterns of description were particularly strong for the parent-child dyads of older children when compared to those of younger children. Older children in our studies may represent more matured members of the culture than younger children in their respective cultural groups, and with these partners, parents may have demonstrated stronger perspectives of their culturally shared attention. However, it should be noted that these older children in our studies have not yet fully learned culturally dominant modes of attention as evident by their solitary performance. Therefore, these older children’s contribution to sustaining culturally shared attention in their community may be limited. Future studies might focus on identifying different sources, such as peers, teachers, and siblings, from which transmission occurs as a means to sustain cultural beliefs and influence the development of culturally dominant modes of attention.

### Limitations and Future Research

The current study has several limitations. First, although we demonstrated that parent–child joint activities facilitated children’s accommodation of culturally dominant modes of attention, the current experimental design did not fully test how much the children internalized and stabilized the respective modes of attention. It is also possible that children may have benefitted from the learning effect in the joint description condition. Also, parents were in the same room as children when they completed the solitary phase. It is important to investigate these issues more comprehensively by modifying the current paradigm. For example, applying the A-B-A´ design [78] to a similar attention task, future research may investigate how children engage in the solitary description task alone first, and then have the children and their parents jointly watch another vignette and discuss their observation together while the vignette is played. At the end of the vignette, only the children would describe the content without any help from their parents. Such a paradigm may also provide an insight into how children internalize the culturally dominant modes of attention. Second, the current research recruited children and parents cross-sectionally, and therefore we could not depict how children gradually develop the culturally dominant mode of attention. To answer this question, a longitudinal examination will be necessary to strengthen our assertion by investigating the process of cultural learning and how the parent–child relationship might change over time [79]. Finally, although the current study attempted to simulate everyday interactions that parents and children engage in by using the joint description task, the participants engaged in experimentally controlled activities in a quiet room, separated from their daily activities. Although this control was necessary to minimize potential confounding variables across cultures, the participants’ experience was not naturalistic. Future research could complementarily mitigate this weakness in the experimental design by investigating cultural learning processes within more naturalistic interactions such as storytelling, reading books, watching movies, and remembering various events together (e.g., [48–49]). Overall, such future investigations would result in a more comprehensive understanding of the parental role in cultural learning processes.

Despite the limitations, our results provide one of the first empirical demonstrations of how parents communicate to their preschool and school-age children in culturally dominant modes of attention, and how children’s cognition develops in such a rich environment with a full of social interactions. Our findings may be helpful to further understanding how cross-cultural differences in attention are sustained over generations, and particularly children learn culturally unique modes of attention.

## Supporting Information

S1 Data The complete data set from both Study 1 and 2. (SAV)

S1 Text Description of the dataset. (DOCX)
