# Supplementary material for: The Communication of Culturally Dominant Modes of Attention from Parents to Children: A Comparison of Canadian and Japanese Parent-Child Conversations during a Joint Scene Description Task
Source: PLoS One. 2016 Jan 29;11(1):e0147199. doi: 10.1371/journal.pone.0147199 (PMC4733050; doi:10.1371/journal.pone.0147199)
Supplement: S1 Text — (DOCX) [file pone.0147199.s002.docx]

Description of the Dataset: PloS_SenzakiMasudaTakadaOkada

**Study**

The study types (1 = Study 1, 2 = Study 2)

**Pp#**

Participants ID number (1000~ = Canadian, 2000~ = Japanese)

**Culture**

Participants’ cultural groups (1 = Canada, 2 = Japan)

**Include**

Participants included and excluded from the final analysis (see the MS for the exclusion criteria, 1 = included, 2 = excluded)

**Age**

Children’s ages

**AgeGroup**

Age group division (1 = 4-6 year-olds, 2 = 7-9 year-olds)

**Gender**

Gender of the children (1 = boys, 2 = girls)

**MotherEd**

Children’s mothers’ education levels (0 = no high school, 1 = obtained high school diploma, 2 = obtained an associate degree, 3 = completed some college classes, 4 = obtained a bachelor’s degree, 5 = post-college degree)

**FatherEd**

Children’s fathers’ education levels (0 = no high school, 1 = obtained high school diploma, 2 = obtained an associate degree, 3 = completed some college classes, 4 = obtained a bachelor’s degree, 5 = post-college degree)

**ParentGender**

Gender of the participating parents in Study 2 (1 = mother, 2 = father)

**Turns**

Numbers of turns taken during parent-child interaction in Study 2

**Dependent Variables (averaged across 6 test vignettes)**

S1_ChildOnly_Total

S1_ChildOnly_Focal

S1_ChildOnly_Back

S1_ChildOnly_Active

S2_Parent_Total

S2_ Parent _Focal

S2_ Parent _Back

S2_ Parent _Active

S2_ChildOnly_Total

S2_ChildOnly_Focal

S2_ChildOnly_Back

S2_ChildOnly_Active

S2_Child_ Parent _Total

S2_Child_ Parent _Focal

S2_Child_ Parent _Back

S2_Child_ Parent _Active
